# Floral nectar microbial communities exhibit seasonal shifts associated with extreme heat: Potential implications for climate change and plant-pollinator interactions

**DOI:** 10.3389/fmicb.2022.931291

**Published:** 2022-08-25

**Authors:** Kaleigh A. Russell, Quinn S. McFrederick

**Affiliations:** Department of Entomology, University of California, Riverside, Riverside, CA, United States

**Keywords:** flower-microbe-bee symbiosis, floral rewards, extreme temperatures, nectar chemistry, *Penstemon heterophyllus*

## Abstract

Floral nectar contains vital nutrients for pollinators, including sugars, amino acids, proteins, and secondary compounds. As pollinators forage, they inoculate nectar with bacteria and fungi. These microbes can colonize nectaries and alter nectar properties, including volume and chemistry. Abiotic factors, such as temperature, can influence microbial community structure and nectar traits. Considering current climate change conditions, studying the effects of increased temperature on ecosystem processes like pollination is ever more important. In a manipulative field experiment, we used a passive-heating technique to increase the ambient temperature of a California native plant, *Penstemon heterophyllus*, to test the hypothesis that temperatures elevated an average of 0.5°C will affect nectar properties and nectar-inhabiting microbial communities. We found that passive-heat treatment did not affect nectar properties or microbial communities. *Penstemon heterophyllus* fruit set also was not affected by passive-heat treatments, and neither was capsule mass, however plants subjected to heat treatments produced significantly more seeds than control. Although we conducted pollinator surveys, no pollinators were recorded for the duration of our experiment. A naturally occurring extreme temperature event did, however, have large effects on nectar sugars and nectar-inhabiting microbial communities. The initially dominant *Lactobacillus* sp. was replaced by *Sediminibacterium,* while *Mesorhizobium,* and *Acinetobacter* persisted suggesting that extreme temperatures can interrupt nectar microbiome community assembly. Our study indicates that the quality and attractiveness of nectar under climate change conditions could have implications on plant-pollinator interactions.

## Introduction

Plants entice pollinators with visual displays, floral scents, and food rewards. A main source of pollinator attraction is floral nectar (Heil, 2011). Natural selection for pollinator attraction shapes floral nectar into complex collections of many chemical and biotic components. Nectar components vary greatly depending on individual plant and even type of nectary, and these changes can affect pollinator foraging and plant fitness (Cnaani et al., 2006). Nectar secretion may also be mediated by abiotic factors experienced by the plant including water availability, light, and temperature (Petanidou and Smets, 1996; Waser and Price, 2016). Nectar is therefore a dynamic solution that contains not only sugars, but also amino acids, proteins, minerals, secondary compounds, and microbial communities that give nectar scent and color (Adler, 2000; Raguso, 2004; Hansen et al., 2007; Nepi et al., 2012; Afik et al., 2014; Rering et al., 2018).

As pollinators visit flowers to forage for nectar, they insert their microbe-covered mouthparts into the nectar, effectively inoculating the nectar with bacteria and fungi (Hausmann et al., 2017). Although nectar properties may make colonization difficult, specifically the osmolarity associated with high sugar concentrations (Pusey, 1999; de Vega et al., 2009), certain microbes are specialized to this ephemeral environment and thrive in the nectar, metabolizing sugars and other components (Herrera and Pozo, 2010; Vannette et al., 2013; Schaeffer et al., 2015). During microbial colonization, the composition of nectar properties changes. Microbes alter sugar concentrations, change amino acid and secondary metabolite composition, and release volatile organic compounds (Vannette et al., 2013; Vannette and Fukami, 2016; Rering et al., 2018; Russell and McFrederick, 2021). Pollinators often choose nectar inhabited by microbes over sterile nectar (Pozo et al., 2012). However, nectar-inhabiting microbial taxa is important to pollinator foraging preference and certain microbes can deter pollinator visitation (Vannette et al., 2013). Therefore, understanding the relationship between nectar properties and microbial colonization is important for pollinator research. However, nectar production and associated microbes are not only influenced by plant physiology and pollinator interactions, but also abiotic factors (Petanidou and Smets, 1996; Tucker and Fukami, 2014; Waser and Price, 2016).

Abiotic factors, such as temperature, have become increasingly recognized as important when studying ecosystem processes, especially in light of global climate change. Global surface temperatures have increased 0.85°C over the past century (Keohane and Olmstead, 2016). According to high CO_2_ emission models, it is predicted that there will be an estimated 5.5°C rise in the United States by the turn of the century (NRC, 2006). Extreme climatic events, including drought and heat waves, are also predicted to become more common (Diffenbaugh et al., 2017). As the frequency of extreme temperature events increase, species interactions and ecosystem functions, such as pollination, may be disrupted (Ockendon et al., 2014). Currently there is a lack of information on how climate change will influence nectar-inhabiting microbial communities and overall nectar attractiveness to pollinators.

In this time of climate crisis and rapid declines in pollinator populations, understanding the effects of warming on nectar-inhabiting microbial communities will give insight into changes in quality of an important food source for insect pollinators – nectar. In a manipulative field experiment, we used passive heating to increase the temperature experienced by a California native plant, *Penstemon heterophyllus*. *Penstemon heterophyllus* has long tube-like flowers which are attractive to many pollinators including hummingbirds, Lepidoptera, and many bee species (Lara and Ornelas, 2008; Pawelek et al., 2009; Salas-Arcos et al., 2017). Because microbes have evolved temperature ranges for optimal growth, we hypothesized that elevated temperature will alter the microbial community structure within nectar. We further hypothesized that because temperature increases can influence nectar-inhabiting microbial communities and plant physiology, this will lead to altered nectar chemistry, leaving the nectar properties dissimilar from those found under ambient conditions.

## Materials and methods

### Plant species and study site

*Penstemon heterophyllus* (Plantaginaceae), the foothill beardtongue, is a drought tolerant, perennial plant that is endemic to the California coastal mountain ranges and Sierra Nevada foothills (Everett, 1950). The flowers of *P. heterophyllus* bloom from April–July, with a lobed, tube-like corolla ranging from 2.4–3.8 cm long. Flowers range from blue to purple and can change color throughout the season. We used the cultivar “Margarita BOP” which has been cultivated for Mediterranean-climates. All plants were obtained from A & F Growers INC. Riverside, CA, and purchased as young plants in 1-gallon pots. A & F Growers is a native plant nursery therefore, our experimental plants were initially grown surrounded by other native plants and potentially visited by pollinators before the purchase date. Our experiment took place in “Ortega Park,” located at the University of California, Riverside’s Agricultural Operations (33°57′48.98” N, 117°20′29.30” W). Plants were immediately placed in Ortega Park for the experiment upon purchase. Ortega Park consists of *Jacaranda* sp. trees and ornamental grass with no other vegetation within 10 m on all sides. The closest vegetation surrounding the park is wild growing *Brassica* sp. plants on one side and cultivated citrus trees on the other sides.

### Experimental design

We set up 25 wooden pallets in “Ortega Park,” a shaded section under Jacaranda trees in UCR’s Agricultural Operations. Five pallets in a row made up one plot with 5 m distance between adjacent pallets. We established a total of 5 plots, each 10 m away from the other. A single pallet consisted of two *P. heterophyllus* plants, one of which was subjected to a passive heating treatment, and one was subjected to a non-heated control (Supplementary Figure 1). All plants were of similar age and had begun flowering when the experiment began. In total, we used 50 plants, 25 in the passive heating treatment and 25 in the control treatment. A passive heating treatment consisted of two 12′ × 12′ Lexan plexiglass, 2 mm thick sheets attached at the edge to create a 90° angle as described in the International Tundra Experiments (ITEX; Marion, 1996). We placed 1-gallon potted plants on the pallet and then surrounded the plant with either a heat-treatment or a control. We placed Hobo data loggers (Onset Computer Corporation, Bourne, MA, United States) on the soil of the pots in both passive heating and ambient control treatments to assess difference in temperature based on treatment. To maximize solar heat, we arranged passive heating treatments around the plants in a south facing direction. We constructed control treatments using Tulle fabric (Joann Item # 15274541) and 18′ wooden dowels. These controls were constructed to form a 90° angle around the plants to act as a physical barrier, similar to that of the heat treatments, but without increase solar radiation and allowing for air flow. We choose this style of heat treatment, opposed to an open top chamber, to heat the plant and allow for pollinator visitation. This set up could have also deterred pollinators, however, we constructed the controls as to allow equal opportunity for pollinators to visit both treatment and control plants. As there was no precipitation during our experiment we watered each plant daily with 0.5 L of water by hand.

### Collection methods

We collected nectar twice a week (on Saturdays and Wednesdays) for 6 weeks in June–August 2018. Occasionally it took more than a day to collect all the samples and collections spilled over into Sundays and Thursdays. Nectar was collected using a 20 μl Biohit^®^ pipette (Swedesboro, NJ) and 20 μl Gilson^®^ pipette tips (Middleton, WI). To quantify nectar production, we recorded volume as we extracted nectar from each flower using volume calibrated pipette tips. Individual flower longevity was not recorded, however, *Penstemon* sp. flowers are known to live 6–15 days (Salas-Arcos et al., 2017), therefore, nectar was collected from individual flowers more than once. We pooled nectar from a single plant into 50 μl of UV sterilized nanopure water in a sterilized and labeled 1.5 ml microcentrifuge tube. We kept pooled nectar on ice in the field and aliquoted each sample upon return to the lab, one portion to analyze nectar sugars and one portion to characterize the microbial communities. To read nectar sugar concentrations we used an Eclipse^®^ hand-held refractometer which reads total percent sugar (Brix%). To account for the additional water that the nectar was collected into we calculated a dilution factor using total nectar collected plus 50ul of water, then divided by total nectar collected. We then multiplied the Brix reading by the dilution factor to get total percent sugars. At the end of the flowering season, we collected capsules from each plant and after a week weighed them in the lab. Once capsule mass was recorded, we dissected the capsules and counted individual seeds. We used these data to analyze fruit set, capsule weight per treatment, and seed set per flower.

We conducted pollinator surveys between 9:00 and 11:00 am twice a week for the duration of the flowering period. We conducted our pollinator surveys at this time because it has been reported as a highly active time for many insect pollinators (Rader et al., 2013). We observed each plot for 30 min and collected all invertebrate floral visitor by slowly hand netting as to disturb the focal plants as little as possible, as typical sweep netting can damage the plant. For any vertebrate visitor, such as humming birds, we recorded number of visits per unit time to each plant.

### Recording air temperature

To measure air temperature, we used the weather station at the University of California, Riverside’s Agricultural Operations Station. We recorded day-time high temperatures ranging from 27 to 43°C. The 43°C temperature, which was recorded on July 7, is an unusually high, one-time temperature event for this area. We are therefore classifying this day as “an extreme temperature event” for the following reasons: (1) it represents the hottest day of the 2018 summer recorded in this area according to weather station data, and (2) in the 2 weeks before and after this one-day event the temperatures were 5–15°C degrees cooler, making it a short-term extreme temperature event. We then broke down our sample collection period into 3 parts based around this extreme temperature event, with the “early collection period” representing the first 4 sampling days, “middle collection period” representing days 5–7 of sampling (collection day 5 was the extreme temperature event), and the “late collection period” representing sampling days 8–11.

### DNA extractions and sequencing

To extract DNA from the pooled nectar samples, we used the manufacturer protocol for TRIzol^®^ Reagent DNA extractions from Life Technologies (Carlsbad, CA). We included four no-template control ‘blank’ samples that contained no nectar sample and that we included in all downstream analyses to control for reagent contamination. To characterize the microbial communities within nectar, we followed the protocols detailed in McFrederick et al. (2016), and used a dual-index inline barcoding to prepare samples for sequencing on the MiSeq sequencer (Illumina, San Diego, CA). We used the bacterial 16S rRNA sequence primers 799F-mod3 CMGGATTAGATACCCKGG (Hanshew et al., 2013) and 1115R AGGGTTGCGCTCGTTG (Kembel et al., 2014) to amplify V5-V6 region of the 16S rRNA gene and the fungal internal transcribed spacer (ITS) primers ITS1F (5’-CTTGGTCATTTAGAGGAAGTAA-3′) and ITS4R (5′ -TCCTCCGCTTATTGATATGC-3′). Both sets of primers included the Illumina sequencing primers, a unique 8-nt-long barcode, and the forward or reverse genomic oligonucleotide (Kembel et al., 2014). We performed PCRs using 10 μl of 2 × Pfusion High-Fidelity DNA polymerase (New England Biolabs, Ipswich, MA), 10 μl of ultrapure water, 0.5 μl of each 10 μM primer stock, and 4 μl of DNA, with an annealing temperature of 57°C for 30 cycles. To remove unincorporated primers and dNTPs, we cleaned the PCR products using Ultraclean PCR cleanup kit (MoBio, Carlsbad, CA). To complete the Illumina sequencing construct, we used 1 μl of the clean PCR product as a template for a second PCR, using HPLC-purified primers: CAAGCAGAAGACGGCATAC GAGATCGGTCTCGGCATTCC TGC and AATGATACGGCGACCACCGAGATCTA CACTC TTTCCCTACACGACG (Kembel et al., 2014). We then normalized 18 μl of PCR product using SequalPrep Normalization plates (Thermo Fisher Scientific, Waltham, MA). We then pooled 5ul of each sample in order to perform another Ultraclean PCR cleanup on this combined normalized PCR product. We assessed library quality using a 2,100 Bioanalyzer (Agilent, Santa Clara, CA). After quality control, we sequenced the libraries using a MiSeq sequencer (Illumina) and MiSeq Reagent kit, version 3 (Illumina), with 2 × 300 cycles, at the IIGB Genomics Core, UC Riverside.

### Bioinformatic analysis

To process the 16S rRNA gene sequence libraries and trim low-quality ends off the reads, we used QIME2-2018.6 (Bolyen et al., 2018). Next, we binned our sequences into amplicon sequence variants (ASVs) using DADA2 (Callahan et al., 2016), followed by removing chimeras and reads with more than two expected errors. We used the q2-feature-classifer (Bokulich et al., 2018) trained to the 799–1,115 region of the 16S rRNA gene to assign taxonomy to the ESVs and conducted local BLASTn searches against the NCBI 16S microbial database (October 8, 2019). We filtered out ASVs from the resulting feature table that corresponded to contaminants of reagents as identified in our blanks along with chloroplast and mitochondria. We used the MAFFT aligner (Katoh and Standley, 2013) and FastTree v2.1.3 (Price et al., 2010) to generate a phylogenetic tree of our sequences.

### Statistical analysis

We used generalized linear mixed models (GLMMs; package lme4) with Poisson error distribution to assess differences in nectar properties by plant through the sampling period in R 3.4.4 (R Core Development Team, 2017). We used nectar sugar concentration or nectar volume as the response variable, temperature treatment as fixed effect, and plant, location in plot nested in plot, collection date, of which there were 11, number of flowers pooled, environmental temperature, and collection period as random effects. To analyze seed characteristics, we used GLMM with Gaussian error distribution, with capsule weight or seed number as the response variable, temperatures treatment as fixed effect and location in plot nested in plot and plant as random effects. We used package lmerTest to compare coefficients of fixed effects.

To assess the microbial communities, we used the phylogenic tree developed from our sequences and ASV table to calculate UniFrac distance matrices. We used the Shannon Diversity Index and linear mixed models to analyze alpha diversity. We used Shannon Diversity Index as the response variable, temperature treatment as fixed effect, and plant, location in plot nested in plot, number of flowers pooled, environmental temperature, and collection period as random effects. We used structural equation models (SEMs; package lavaan) to describe direct and indirect relationships between nectar microbial community with nectar proprieties and seed set. We expected to find evidence of microbial diversity directly affecting nectar volume, sugars, and seed set and an indirect effect of temperature on these variables. We therefore set up our model with 1 endogenous latent variable describing nectar properties (volume and sugar concentration), 2 endogenous variables describing seed set and microbial diversity (Shannon’s diversity index), and 1 exogenous variable describing the environmental conditions (daily temperature).

For analyses of beta diversity, we first used betadisper to test for homogeneity of dispersion, then used Adonis (999 permutations PERMANOVA) both in the R-package vegan (Oksanen et al., 2008) with treatment, environmental temperature, and collection period as independent variables and the Generalized UniFrac matrix as the dependent variable. We also used the Generalized UniFrac distance matrix to perform principal coordinate analysis (PCoA) with treatment and collection period as covariates. We performed a non-parametric microbial interdependence test (NMIT) to determine longitudinal sample similarity as a function of temporal microbial composition, in QIIME2 using temperature treatment as the subject. We then performed a feature volatility analysis from q2-longitudinal, to identify indicator species with changes in temperature due to treatment. We performed a time series analysis using the Bray-Curtis distance matrix to determine within group community differences, then compared these differences across collection period using a one-way analysis of variance (ANOVA).

## Results

### Nectar properties

**Figure 1:**
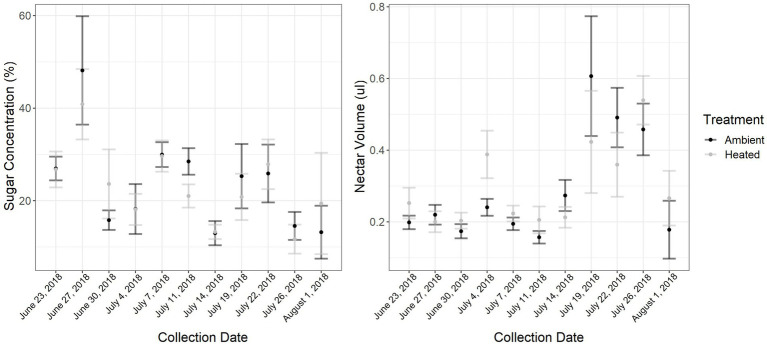
Nectar sugar concentrations per plant (nectar was pooled for each plant) throughout the sampling period (6 weeks), was significantly influenced by collection date and environmental temperature. Nectar volume per plant throughout the sampling period was significantly affected by collection date as well. The extreme temperature event occurred July 7 where the maximum temperature was 43.5°C, a 9.5°C increase from the previous day. Error bars represent standard error.

ITEX passive heating features successfully increased day-time temperatures with a range of 0°–12.8°C above control treatment with an average increase of 0.58°C on heat treatment plants (Supplementary Figure 2). The ITEX heating system is known to only increase day-time temperatures with little to no effect on night-time temperatures (Marion et al., 1997) and heating can be very variable. For example, if the focal plant was in direct sun the increase could be as high as 12.8°C, while a plant that had partial shade at the same time would exhibit lower heating. There were also occasions where the control read slightly higher temperatures than heated treatments. This usually happened in the mornings when direct sunlight was on the control plant for a short period of time. Ambient day-time high temperatures ranged from 28.5 to 43.5°C, with an extreme temperature event which began July 7 (collection day 5). We collected a total of 550 nectar samples: 200 in the early collection period, 150 in the middle, and 200 in the late collection period. Nectar volume was influenced by collection date (GLMM: F_1,8_ = 5.0581, *p* < 0.001; Figure 1), specifically the middle collection period when the extreme temperature event occurred (GLMM:F_1,91_ = −2.4, *p* = 0.01) but not temperature treatment (GLMM: F_1,44_ = 1.3752, *p* = 0.25), with overall mean of 6.4 ± 0.29 (SE) μl of nectar from control and 5.12 ± 0.29 (SE) μl from heat treatment plants. Generally, there was more nectar in the ambient treatment in the late collection period when overall temperatures were higher, but there were some collection days in the early collection period where more nectar was extracted from the heated plants. Nectar sugars were influenced by collection date (GLMM: F_1,91_ = 3.85, p < 0.00; Figure 1), specifically the middle and late collection period (GLMM:F_1,91_ = −3.4, p < 0.001; GLMM:F_1,91_ = −4.2, p < 0.001, respectively) and environmental temperature (GLMM:F_1,91_ = 3.4, p < 0.001). However, there was no effect of temperature treatment (GLMM: F_1,44_ = 0.0972, *p* = 0.7567). There were no recorded floral visitors on the focal plants during the pollinator surveys.

### Seed set

Capsule mass (F_1,16_ = 0.15, *p* = 0.702; Supplementary Figure 3) and number of capsules per plant (F_1,20_ = 1.1, *p* = 0.304), were unaffected by temperature treatments with an average weight of 17.5 ± 0.8 mg in the control treatment and 16.2 ± 0.9 mg in the heat treatment. There were significantly more seeds in the capsules of flowers from plants that were subjected to the heated treatment (F_1,5_ = 4.89, *p* = 0.019; Supplementary Figure 4), with an average of two more seeds per capsule than the ambient treatment, with 9.7 ± 0.7 and 7.7 ± 0.017 average seeds per capsule, respectively. However, this average is likely due to the handful out outlier capsules that had up to 40 seeds.

### Microbial communities

**Figure 2:**
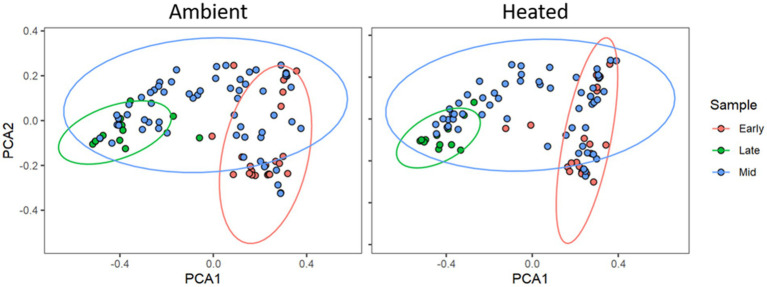
Principal Coordinates Analysis plot of the Generalized UniFrac distance matrices of microbial communities in P. heterophyllus in both treatments through time. Red points indicate the microbial communities for the early collection period (June 23, 2018 -July 5, 2018), blue points denote the middle (mid) collection period (July 7, 2018 -July 12, 2018) and green points indicate the late collection period (July 14, 2018 -August 1, 2018). Colored ellipses designate 95% confidence intervals around the centroid median of the points. Adonis analysis indicated significant dissimilarity of microbial communities in P. heterophyllus flowers through time. (*F*_1,189_ = 9.8, *R*^2^ = 0.172, *p* = 0.001).

**Figure 3:**
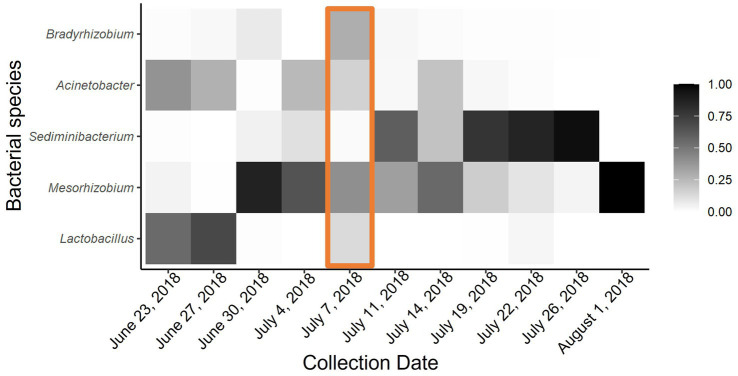
Heat map of proportional abundance of five most dominant bacterial taxa across all collection points. Adonis analysis indicated significant dissimilarity of microbial communities in *P. heterophyllus* flowers due to environmental temperature. Orange box indicates extreme temperature event.

There was a total of 2,157,069 quality-filtered reads with an average of 5,104 reads per sample that clustered into 284 filtered exact sequence variants (ASVs). Through rarefaction analysis, we determined that we had representative coverage of bacterial species diversity at a depth of 2000 reads per sample. We were not able to amplify or sequence fungi from any of our samples. Using the Shannon Diversity Index, we found no significant difference in alpha diversity between temperature treatments (F_1,268_ = 0.3481, *p* = 0.55). However, there were significant differences between communities due to environmental temperature (F_1,268_ = 14.604, *p* = 0.02). PCoA analysis on the Generalized UniFrac distance matrix (Figure 2) showed clustering by time in both the two-dimensional ordinations. We analyzed the Generalized UniFrac distance matrix of our samples with Adonis and found no significant difference between heating treatments (F_1,189_ = 0.322, *R^2^* = 0.00157, *p* = 0.633), however there was a significant effect of environmental temperature (F_1,189_ = 16.122, *R^2^* = 0.078, *p* = 0.001; Figure 3), and collection period (F_1,189_ = 9.8, p = 0.001). There was no effect of microbial of microbial communities on nectar properties (*z* = −0.911, *df* = 7, *p* = 0.363) or seed set (*z* = 0.417, *df* = 9, *p* = 0.677) according to structural equation models. For the nectar microbiomes through time, dispersion was significantly heterogenous between early, middle, and late collection periods (*p* < 0.001, F_2,196_ = 65.07). The non-parametric microbial interdependence test also found no significant difference by heating treatment (NMIT = 1.22, *p* = 0.06).

Although there was no significant difference between microbial communities due to the passive heating treatments, there was significant bacterial turnover throughout the duration of our experiment. Notably, time series analyses show that microbial communities in both the heated and non-heated treatments experience similar shifts throughout the season (Heated: F_1,94_ = 57.24, *p* < 0.00; Control: F_9,86_ = 26.59, *p* < 0.00). Specifically, during the early collection period the microbial community was dominated by *Lactobacillus, Mesorhizobium,* and *Acinetobacter* (Figure 3). After the extreme temperature event there was a loss of *Lactobacillus* and new colonization by *Sediminibacterium*, while *Mesorhizobium* and *Acinetobacter* were still present (Figure 3). By the late collection period *Sediminibacterium* dominated the nectar microbial community but with *Mesorhizobium* and *Acinetobacter* still present (Figure 3). Feature volatility analysis confirmed these qualitative patterns by revealing that *Lactobacillus, Sediminibacterium, and Acinetobacter* were the indicator species that corresponded with change in temperatures (24, 6, and 3% importance respectively).

## Discussion

Our data revealed longitudinal shifts in nectar properties and nectar-inhabiting microbial communities across the flowering season. The most drastic shift correlated with an extreme temperature increase, while our passive heating treatment had no detectable effect on nectar properties or microbial communities. At collection day #5, July 07, 2018, the immediate area experienced a 9.5°C temperature spike, with a high of 40°C recorded on our temperature logger data and 43°C recorded air temperature. Interestingly, it was at this time point that we observed a shift in microbial communities followed by changes in nectar properties (volume and sugars). Extreme temperatures are predicted to become increasingly common under climate change scenarios (Diffenbaugh et al., 2017), and our data suggests that these extreme events may act as an environmental filter on the nectar microbial communities. This is common in soil systems, where extreme temperatures, such as fire, will select for heat tolerant colonizers after the event (Hinojosa et al., 2016).

Nectar properties were not affected directly by temperature treatments but did change across time. Although our data do not link shifts in nectar properties with changes in the microbial community, there is ample documentation that microbial communities can alter nectar properties (Vannette et al., 2013; Russell and McFrederick, 2021). As increases in temperature affect microbial community composition, extreme temperature events could indirectly affect pollination services. Changes in nectar properties resulting from microbial inoculation can influence pollinator preference (Vannette and Fukami, 2016) and potentially pollination services and plant fitness (Pozo et al., 2014). Conversely, many penstemon species are able to rapidly refill nectar upon pollinator consumption (Castellanos et al., 2002). High temperatures can increase rate of water evaporation in the nectar as it is being replenished (Villarreal and Freeman, 1990). This evaporation will change the water and sugar concentrations in the nectar, potentially making it less suitable for nectar specialists to colonize (Pusey, 1999; de Vega et al., 2009). Microbial community composition throughout the flowering season is therefore important for pollination services and potentially sensitive to extreme temperature changes.

Nectar-inhabiting microbial communities were significantly affected by environmental temperature. There was a shift in community structure with the loss of the initially abundant *Lactobacillus* ASV immediately following the extreme temperature event. The *Lactobacillus micheneri* clade is commonly associated with plants and pollinators and exhibits optimal growth from 30 to 35°C and no growth at 40°C and above (McFrederick et al., 2018). The persistence of *Mesorhizobium* and *Acinetobacter* at low levels in the nectar despite the extreme temperatures suggests they are more equipped to handle these temperature spikes. *Acinetobacter* is an environmental bacterium that is commonly found in nectar worldwide and whose abundance is affected by temperature and other environmental factors (Sharaby et al., 2020). Some strains of *Acinetobacter* and *Mesorhizobium* have performed well in incubation temperatures up to 44°C or when heat shocked at 48°C in laboratory studies, respectively (Laranjo and Oliveira, 2011; Krizova et al., 2015). Overall, our findings suggest that the extreme heat event led to a loss of *Lactobacillus* from nectar, which then became open for colonization by *Sediminibacterium* while *Mesorhizobium* and *Acinetobacter* persisted across the entire collection period, although in low levels.

Priority effects can drive community assembly, especially in nutrient rich and ephemeral nectar-inhabiting microbial communities. In sticky monkey flower (*Mimulus aurantiacus*) nectar, the microbe that initially established dominance was continually found across multiple floral generations (Toju et al., 2018). Furthermore, temperature fluctuations prevented extinction of late-arriving species that initially may have been excluded due to priority effects (Tucker and Fukami, 2014). High temperatures have also been shown to have a negative effect on microbial biodiversity. For example, Sharaby et al. (2020) found that slightly elevated temperatures corresponding to elevation significantly reduced bacterial community diversity and evenness.

Our study extends these previous studies by showing that an extreme temperature event can interrupt community assembly. We initially found communities dominated by *Lactobacillus* which established and persisted for the first couple weeks of sampling until the dramatic temperature increase occurred. This one day of extremely high temperature may have facilitated a shift in the microbial communities, allowing for previously unrepresented taxa to colonize the nectar. Once temperature stabilized these new colonists persisted, resulting in different nectar communities pre- versus post-temperature event. Extreme weather events may therefore disturb nectar microbiome community assembly.

We acknowledge that a caveat to our study is a lack pollinator visitation to our focal plants, which are the usual source of microbial inoculation. This is potentially due to the location of our experimental set up as there was little forage in the area and the presence of surrounding co-blooming plants influences pollinators’ diversity and abundance (Lázaro et al., 2009; Layek et al., 2021). The initial nectar-inhabiting microbial community could have been inoculated into flowers at the nursery of origin. These plants were purchased at a native plant nursery already in bloom and potentially visited by wild pollinators before the beginning of our experiment. Ants were often seen on our flowers later in the day (after pollinator surveys) and could potentially be how microbes persisted within the community. Another possible explanation is that colonist microbes were vectored by the wind (Brysch-Herzberg, 2004; Zemenick et al., 2018). We also acknowledge that we manipulated a small number of plants in the landscape, therefore not controlling for the source pool of fresh microbial inocula coming from nectar of non-experimental plants (plants not heated with the ITEX method). However, due to the observed lack of bee and hummingbird pollinators suggests that temperature can shift nectar-inhabiting microbial communities to more heat tolerant species, opposed to pollinators inoculating the nectar with microbes from the surrounding landscape. We saw a second slightly smaller spike in temperature towards the end of the collection period that was not associated with a change in microbial community further suggesting this initial extreme temperature event early in the season could have selected for more specialized nectar-inhabiting microbes.

Although we saw no changes in microbial community with treatment, we are not able to rule out the effect of temperature on this system. The limitations of the ITEX system are the lack of night-time heating and temporal heterogeneity of the treatment as some plants may have partial shade during the day inhibiting the temperature increase. The value in these results is that small background heating does not appear to disturb nectar-inhabiting microbial communities in this system. Our results may have differed if we had conducted this work in a cooler climate, where the ITEX heating system may have greater effects on temperature treatment. Therefore, we acknowledge that much more work needs to be done in this area to understand how the nuances of climate change will affect nectar-inhabiting microbes and their interaction with pollinators.

As heat treatment may affect the plants themselves, we quantified seed and fruit set at the end of the experiment. Capsule counts per plant and capsule mass were the same between treatments, however the passive heat-treated plants had more seeds than the controls. This may be due to a decrease in seed size in the heat-treated plants, as capsule weight did not change. Seed size can be both directly and indirectly influenced by heat. For example, although *Penstemon* can self-pollinate, there is evidence the seeds production is higher when *Penstemon* sp. are outcrossed with no difference in weight or in percent viability in outcrossed vs. self-pollinated seed (Clements et al., 1999). In forbs, seed size and germination success negatively correlate with high temperatures (Yi et al., 2019) as does seed size and drought tolerance (Martínez-López et al., 2020). Similar effects occur in certain crop plants, where heat can decrease seed size (Folsom et al., 2014). Decreased seed size can be caused by changes in genetic expression in the plant due to heat stress (Folsom et al., 2014; Chen et al., 2016). Seed size can affect plant germination success and potentially plant fitness; it has been well documented that smaller seeds are less competitive than larger seeds (Leishman, 2001). Studies on forbs have observed a positive relationship between seed size and survival from established seedlings to reproduction (Metz et al., 2010). In most cases in forbs, the larger the seeds the more likely the plant is to germinate and survive and the more tolerant the plant is to extreme conditions. If heat treated plants are producing smaller seeds, these seeds could be slower growing and less competitive for resources than larger seeds from the ambient treatment, especially in instances of extreme climatic events.

Future studies are needed to determine how climate change will continue to effect pollination services. Although our study begins to look at subtle daytime warming and temporary extreme temperature events on nectar-inhabiting microbes, the reality of climate change includes rapid night-time warming (DeGaetano and Allen, 2002) which was absent from this study. Plants, pollinators, and associated microbes may interact differently under night-time and whole landscape warming and further studies disentangling these pressures on pollination would be of great importance.

## Conclusion

Although our passive-heat treatments had little impact, an extreme temperature event in the middle of our experiment appeared to have large effects on nectar properties and nectar microbial communities. Specifically, a loss in *Lactobacillus* from nectar communities during the rise in temperature indicated that extreme temperatures can change microbial community structure, allowing new community members to colonize. Although we were unable to obtain pollinator preference data, this may ultimately affect visitation rates and successful pollination. As severe weather events are predicted to become more commonplace as climate change worsens, our data suggest that extreme temperature events could alter plant-pollinator-microbe interactions and further research on this topic is warranted.

## Data availability statement

Metabarcoding amplicon data and associated metadata are available on the NCBI Sequence Read Archive (SRA PRJNA717043).

## Author contributions

KR and QM designed this experimental setup and edited further drafts. KR collected and analyzed the data and wrote the first draft of the manuscript. All authors contributed to the article and approved the submitted version.

## Funding

This work was supported by USDA NIFA pre-doctoral fellowship 2019 -67011-29604, AES Hatch Project CA-R-ENT-5109-H, and the National Science Foundation NSF DEB 1929572.

## Conflict of interest

The authors declare that the research was conducted in the absence of any commercial or financial relationships that could be construed as a potential conflict of interest.

## Publisher’s note

All claims expressed in this article are solely those of the authors and do not necessarily represent those of their affiliated organizations, or those of the publisher, the editors and the reviewers. Any product that may be evaluated in this article, or claim that may be made by its manufacturer, is not guaranteed or endorsed by the publisher.
