# Supplementary material for: Floral nectar microbial communities exhibit seasonal shifts associated with extreme heat: Potential implications for climate change and plant-pollinator interactions
Source: Front Microbiol. 2022 Aug 25;13:931291. doi: 10.3389/fmicb.2022.931291 (PMC9453676; doi:10.3389/fmicb.2022.931291)
Supplement: Supplementary file 3 [file Presentation_1.pdf]

A

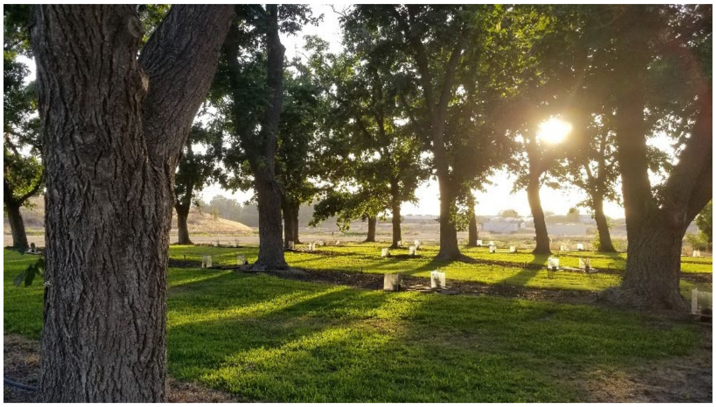

B

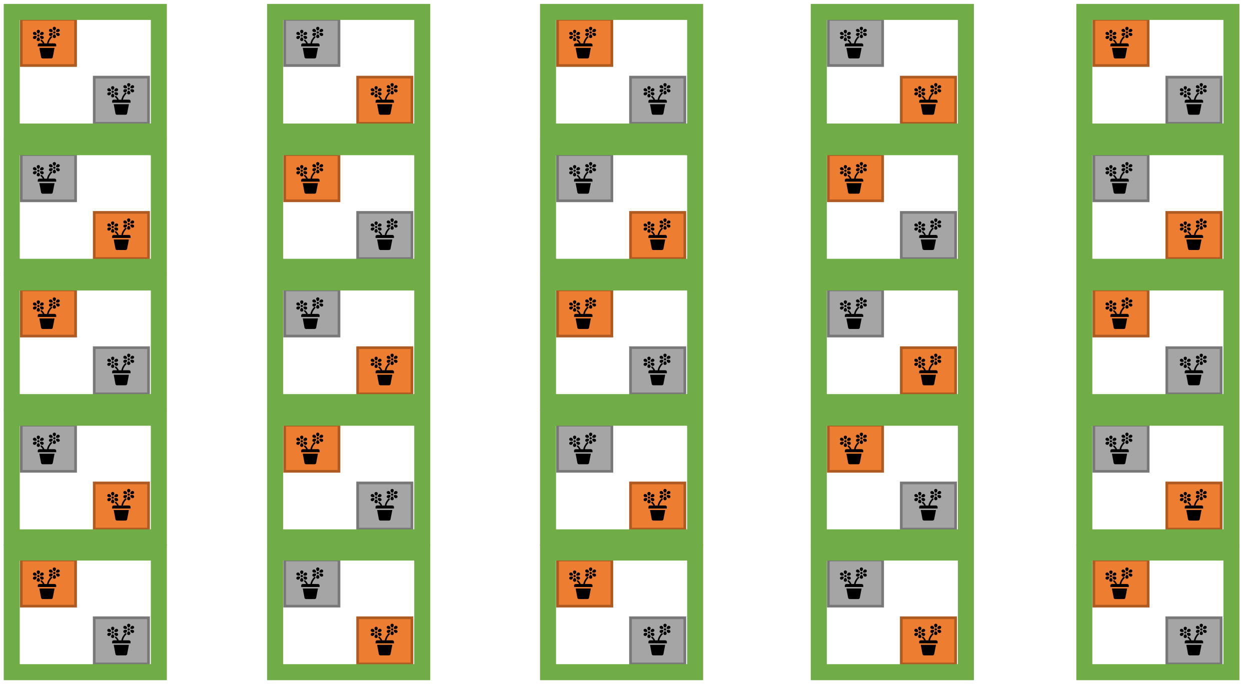

Supplementary figure 1. A) Image of experimental plot in the Agricultural Operations at the University of California, Riverside. B) Design set up. Green squares represent a pallet, orange box represents a heat-treated plant, and gray box represents a control plot. In a “column”, pallets were 5m apart, rows were 10m apart.

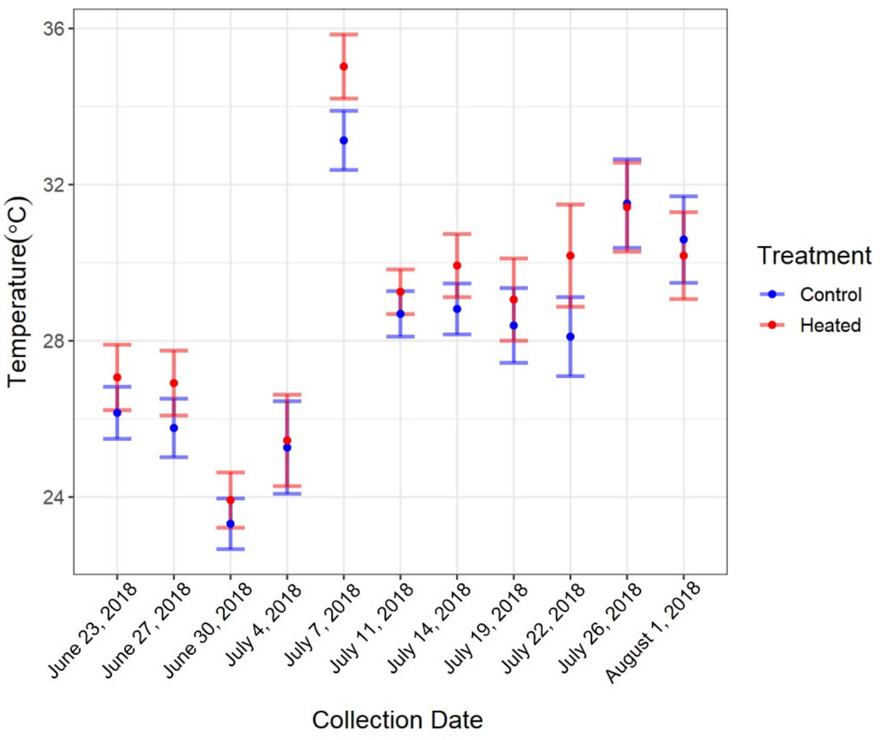

Supplementary figure 2. Recorded day-time temperature in heated and control treatments. Error bars represent standard error.

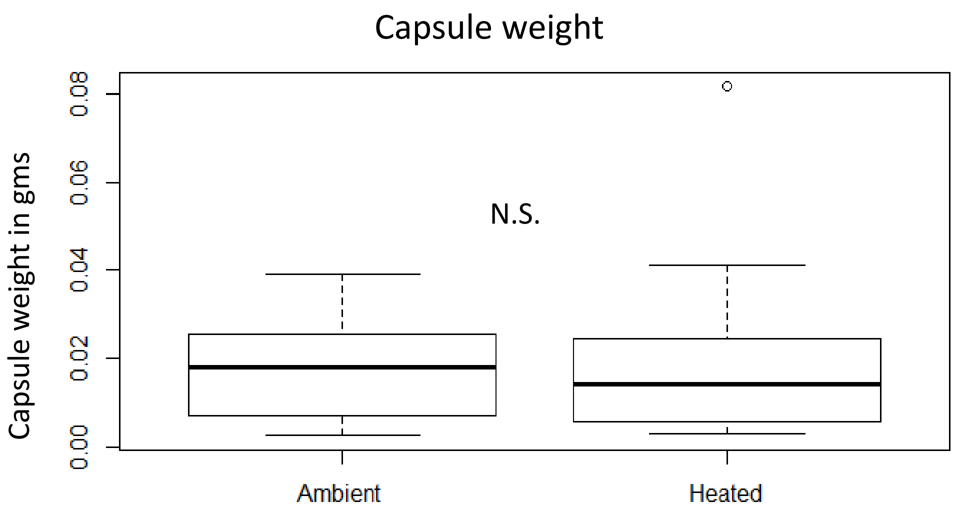

Supplementary figure 3. Mass of capsules did not differ between temperature treatments when collected at the end of the flowering period, after the 7-week collection period ($t=1.5478$, $df=558.94$, $p=0.1222$).

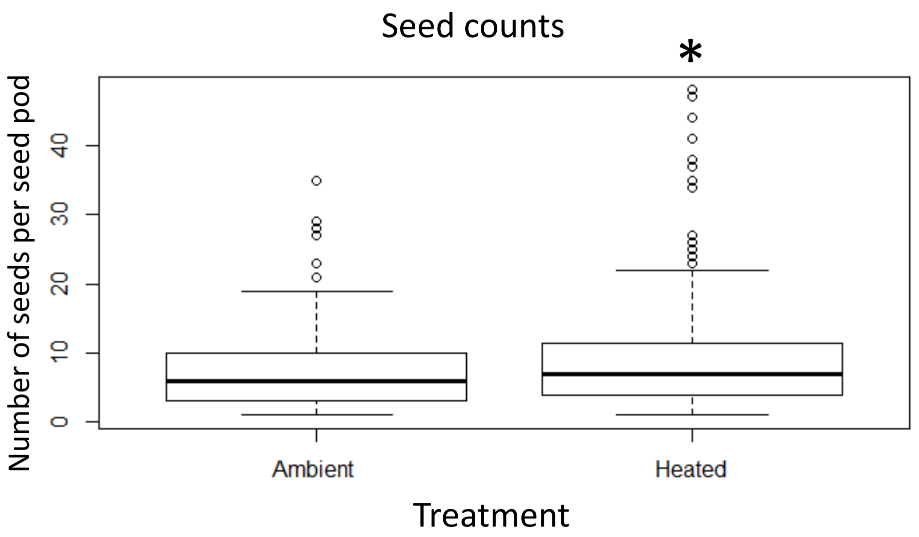

Supplementary figure 4. Number of seeds within the capsule differed by treatment. There were more seeds in the capsules of the heated treatment compared to the ambient treatment ($F_{1,335}=5.482$, $p=0.019$).
